# The Effect of Ureteric Stenting on Female Sexual Function: A Prospective Cohort Study

**DOI:** 10.7759/cureus.11075

**Published:** 2020-10-21

**Authors:** Zehra Kazmi, Daniya Umer, M Hammad Ather

**Affiliations:** 1 Urology, The Aga Khan University, Karachi, PAK; 2 Surgery, The Aga Khan University, Karachi, PAK

**Keywords:** sexual dysfunction, fsfi, fsd, dj stent, jj stent

## Abstract

Background and objective

Urolithiasis is a highly prevalent disease worldwide, with Pakistan belonging to the stone belt of Asia. The usage of the double J (DJ) stent is highly effective when it comes to the management of urolithiasis. However, studies investigating the side effects of DJ stent placement on sexual function in individuals are scarce. In this study, we aimed to assess the impact of DJ stent placement on sexual function in women.

Methods

After obtaining ethical approval, a prospective study was conducted at a university hospital from June 2018 to September 2019. All sexually active women requiring semi-rigid ureteroscopy (URS) or flexible URS [retrograde intrarenal surgery (RIRS)] were enrolled. Women with DJ stent placement (Group A) were compared to women who did not require DJ stent (Group B). The outcome variable was to assess temporary sexual dysfunction after DJ stent placement using the standardized Female Sexual Functionality Index (FSFI) in English or its validated vernacular version. The FSFI was completed at four weeks, and again at three months, following URS/RIRS.

Results

Of the 106 sexually active women initially included in the study, 69 were found to be eligible for final analysis. In Group A, the mean FSFI score at the initial presentation was 31.54 ±4.37. The mean FSFI score at four weeks was lower compared to the baseline score (0 time): 13.96 ±5.5 (p<0.05). At three months, the mean FSFI score returned to near baseline at 32.053 ±5.35 with no significant difference (p=0.65). In comparison to women in Group B, the mean FSFI score at four weeks was significantly lower in Group A (28.87 ±6.59 vs. 13.96 ±5.49; p<0.05). However, there was no significant difference between the mean FSFI scores at any of the three time points within Group B.

Conclusion

DJ stent insertion results in transient postoperative sexual dysfunction in women, which resolves spontaneously within a span of three months after stent removal.

## Introduction

Upper tract urolithiasis is a highly prevalent disease worldwide with prevalence rates ranging from 7 to 13% in North America, 5-9% in Europe, and 1-5% in Asia [1]. For most stones managed by endourological procedures, a double J (DJ) stent is an indispensable device. It offers effective drainage of the urinary tract and is very convenient to use. However, DJ stents are associated with frequent side effects and have a significant impact on the patient’s quality of life [2]. There are many papers published on the impact of an indwelling DJ stent on the patient’s quality of life [3,4]. In addition, contemporary literature also describes various effective treatment modalities to help with stent-related symptoms [5,6]. However, there is a dearth of literature evaluating the impact of DJ stents on sexual function in women.

The aim of our study is to prospectively analyze the impact of an indwelling DJ stent on female sexual function. In most societies, female sexual dysfunction (FSD) remains an issue that is rarely discussed. In this day and age, there is increasing awareness among women regarding sexual health, and they are now more forthcoming when it comes to such sensitive issues. The Female Sexual Function Index (FSFI) [7] has been translated into and validated in the vernacular [8]. It is a 19-segment-based, self-administered questionnaire consisting of six domains, which has been validated for use in diagnosis and categorization of FSD [9].

## Materials and methods

Study design

This prospective cohort study was conducted at the Aga Khan University Hospital in Karachi, Pakistan. The sample size was calculated using the OpenEpi program (version 3), with a power of 80% and a two-sided confidence interval of 95%. The study group consisted of 106 consecutive sexually active female patients presenting to our hospital with a diagnosis of ureteric calculi and requiring ureteroscopy (URS). After obtaining approval from the Ethical Review Committee, all sexually active females presenting with ureteral stone diseases requiring URS were enrolled in the study. They were equally divided into two groups: Group A required stent placement for approximately four weeks postoperatively, while Group B consisted of women who did not require any DJ stent placement. All of these women were followed up prospectively up to a period of three months. The FSFI questionnaire (a previously validated, brief, and self-administered 19-item questionnaire assessing six domains of female sexual function) was used to evaluate FSD. It was specifically designed to assess sexual dysfunction over the preceding four weeks.

Participants

Patients who had a complicated URS {associated with a high-grade injury to the ureter [postureteroscopic lesion scale grade 2+ (PULS 2+)], incomplete stone fragmentation, bilateral stents, Clavien-Dindo grade III + complications}, urinary tract infection (UTI) within four weeks prior to the procedure, pregnancy, previous history of endourological procedures, pelvic surgery or radiotherapy, or those women who were lost to follow-up, were excluded from the study. The indications for stent placement were left to the discretion of the operating surgeon. These included the presence of ureteral wall edema, suspicion of stone migration, impacted stones, or patients from outside the city who required traveling for further follow-up and management.

Data collection

All patients were requested to fill in the FSFI form at the initial visit (time 0), which was taken as their baseline score; the scores were again recorded in two subsequent instances: at four weeks and at three months after the procedure. The outcome variables were to assess the impact of stent placement on sexual function and to compare it between the two groups.

Data analysis

Statistical analysis was performed using Stata/SE 12.1 and Microsoft Excel (Microsoft Corporation, Redmond, WA). Data were stratified quantitatively and descriptively. Quantitative parameters included the FSFI total scores and domain scores for the stent and no-stent groups. These were compared using the Student’s paired t-test, Wilcoxon Rank Sum Test, and chi-squared test as applicable. Descriptive parameters included age, site of the stent, duration of stenting (in days), and the language of the FSFI questionnaire. A p-value of less than 0.05 was considered to be statistically significant.

## Results

A total of 106 sexually active women were initially included in the study. Out of this, 37 women were excluded; the most common reason was the refusal to participate because of hesitation to discuss intimate details. The final study population included 69 women (36 in group A and 33 in group B). Patients were given the option to fill in the FSFI form either in the vernacular or English, based on their preference. Overall, the English form was filled in by 55% of the patient cohort. The average age of women in group A was 42.83 ±10.7 years and that in group B was 45.21 ±12.68 years, and no significant difference was found between the two groups in terms of age (p=0.4). The demographic data of the participants are shown in Table 1. Semi-rigid URS was performed in 47.8% of women in Group A and 33.3% in Group B, whereas retrograde intrarenal surgery (RIRS) was performed in 4.3% of women in Group A and 14.5% in Group B. Using the chi-squared test, a significant association was found in terms of the type of procedure performed between groups, where RIRS was more frequently performed in Group B as compared to Group A (p<0.04).

The average duration of the DJ stent insertion in participants from Group A was 37 days. Table 2 shows a comparison of the domains of sexual function in both groups, and each one of the six domains (desire, arousal, lubrication, orgasm, satisfaction, and pain) showed that there was a significant association between sexual dysfunction and the presence of indwelling DJ stent (p<0.05).

**Figure 1:**
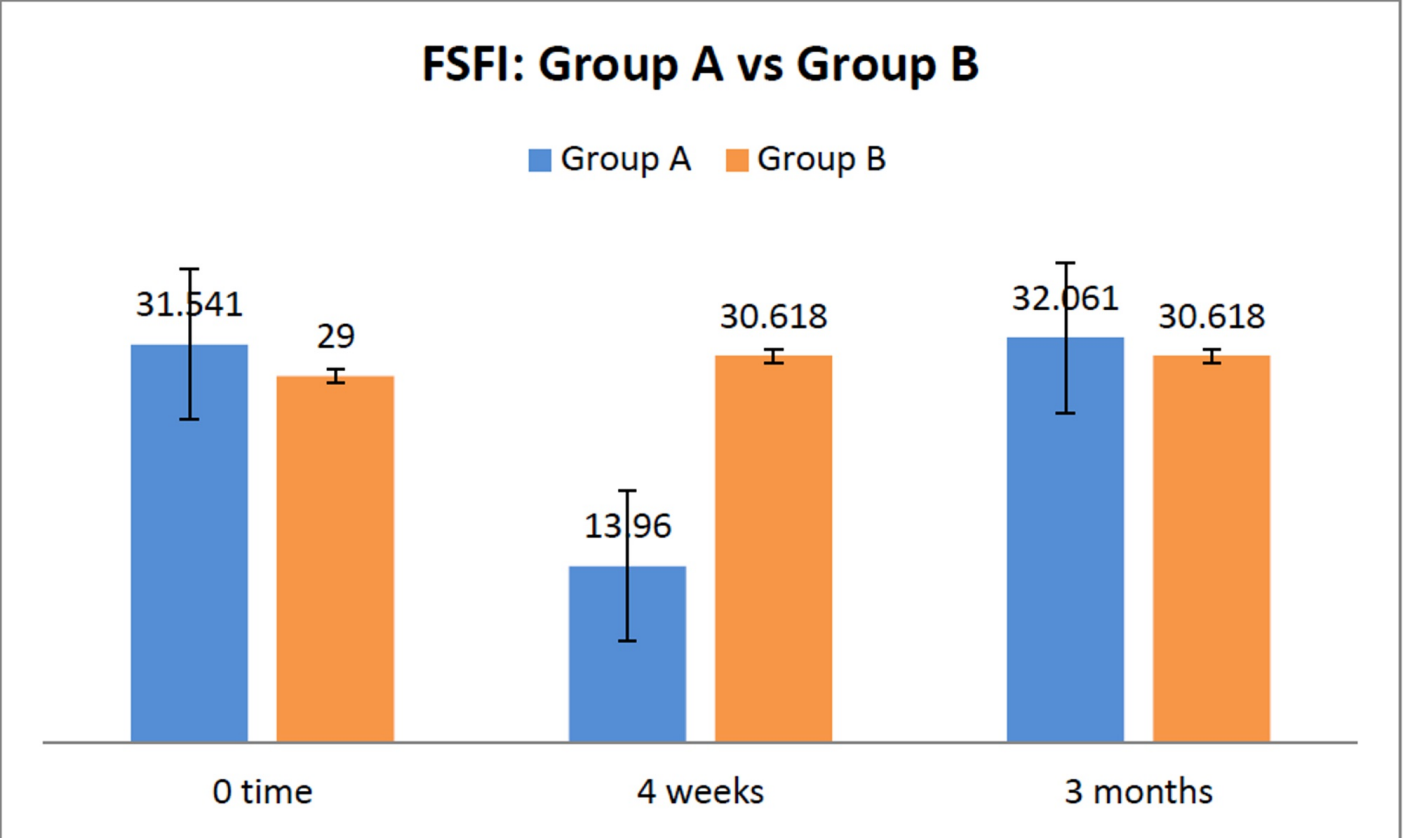
FSFI score before procedure (0 time) and after procedure (at four weeks and at three months) FSFI: Female Sexual Functionality Index

After three months of the procedure, the FSFI score returned to near baseline (0 time) in Group A. In Group A, the mean FSFI score at the initial presentation was 31.54 ±4.37. The mean FSFI score at four weeks was significantly lower compared to the baseline score at 0 time: 13.96 ±5.5 (p<0.05). At three months, the FSFI score returned to baseline at 32.053 ±5.35 with no significant difference (p=0.65). In comparison to women in Group B, the mean FSFI score at four weeks was significantly lower in Group A (28.87 ±6.59 vs. 13.96 ±5.49; p<0.05). However, there was no significant difference between the mean FSFI scores at any of the three time points within Group B (Figure 1).

**Table 1:** Demographic characteristics of the patient cohort Group A: patients with stent; Group B: patients with no stent RIRS: retrograde intrarenal surgery; URS: ureteroscopy; SD: standard deviation

Parameters	Group A	Group B	P-value
Age in years, average ±SD	42.83 ±10.7	45.21 +12.685	0.402
Location of stone: proximal, mid, distal; n (%)	17 (24.6%), 8 (11.6%), 11 (15.9%)	14 (20.3%), 3 (4.4%), 16 (23.2%)	
Procedure: RIRS, URS; n (%)	3 (4.3%), 33 (47.8%)	10 (14.5%), 23 (33.3%)	
Site of stent insertion: right, left; n	21, 15	- -	
Stenting duration in days, average ±SD	37.02 ±12.3	- -	
Language; English, vernacular; n (%)	19 (27.5%), 17 (24.6%)	19 (27.5%), 14 (20.3%)	

**Figure 2:**
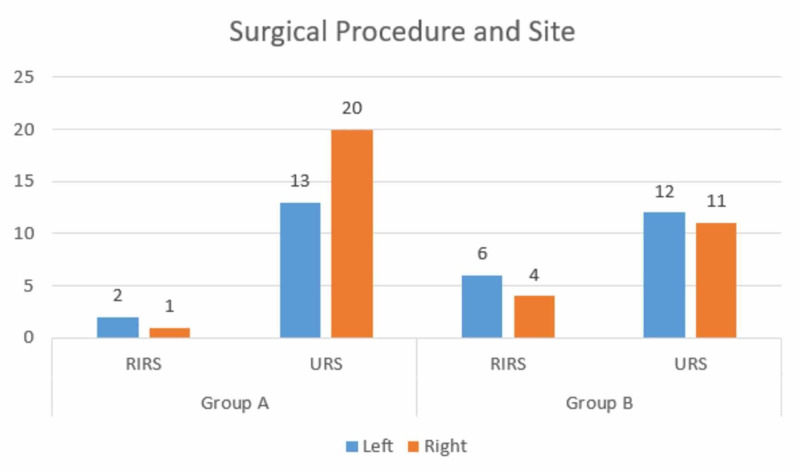
Surgical procedures and sites RIRS: retrograde intrarenal surgery; URS: ureteroscopy

Figure 2 compares the procedures performed with the sites of surgery.

We further evaluated the FSFI scores in a subdomain-based manner including sexual desire (FSFI-SD), sexual arousal (FSFI-SA), sexual lubrication (FSFI-SL), sexual orgasm (FSFI-SO), general satisfaction (FSFI-SS), and pain (FSFI-SP). These scores demonstrated a statistically significant difference (p<0.05) when compared between the two groups at four weeks post-procedure (Table 2). Total FSFI values and subdomains values of the females obtained before and after the stent insertion are presented in Table 3.

**Table 2:** Total FSFI and subdomain scores at four weeks: comparison between Group A and Group B FSFI: Female Sexual Function Index; SD: sexual desire; SA: arousal; SL: lubrication, SO: orgasm; SS: satisfaction; SP: pain; t: total; SD: standard deviation

Domains		Postoperative score (at 4 weeks), mean ±SD	P-value
FSFI-SD	Group A	3.383 ±0.963	<0.05
	Group B	4.18 ±1.05	
FSFI-SA	Group A	2.63 ±1.051	<0.05
	Group B	4.481 ±1.394	
FSFI-SL	Group A	2.225 ±1.730	<0.05
	Group B	4.972 ±1.4792	
FSFI-SO	Group A	1.533 ±1.5667	<0.05
	Group B	4.860 ±1.4735	
FSFI-SS	Group A	2.233 ±1.1084	<0.05
	Group B	5.345 ±0.870	
FSFI-SP	Group A	2.22 ±1.869	<0.05
	Group B	5.030 ±1.319	
FSFI-t	Group A	13.96 ±5.5	<0.05
	Group B	30.618 ±7.027	

**Table 3:** Total FSFI and subdomain scores before and after DJ stent insertion (Group A) FSFI: Female Sexual Function Index; SD: sexual desire; SA: arousal; SL: lubrication, SO: orgasm; SS: satisfaction; SP: pain; t: total; SD: standard deviation

Domains	Preoperative (0 time), mean ±SD	Postoperative (at 4 weeks), mean ±SD	P-value
FSFI-SD	4.683 ±0.96	3.383 ±0.963	<0.05
FSFI-SA	5.4905 ±0.9206	2.63 ±1.051	<0.05
FSFI-SL	5.383 ±0.709	2.225 ±1.730	<0.05
FSFI-SO	5.355 ±1.0530	1.533 ±1.5667	<0.05
FSFI-SS	5.44 ±0.845	2.233 ±1.1084	<0.05
FSFI-SP	5.611 ±0.5775	2.22 ±1.869	<0.05
FSFI-t	31.541 ±4.3705	13.96 ±5.5	<0.05

## Discussion

Interventions for ureterolithiasis depend upon many factors: location, size and stone burden, symptomatic status, and failure of conservative measures. URS is a commonly performed intervention in the management of ureteral stones [10,11]. Endourological interventions for the ureter are often accompanied by the placement of DJ stents in order to decrease complications related to stone fragmentation, to allow better drainage of the kidney, and to decrease the need for pain killers following URS [12]. DJ stent insertion may have a significant impact on the patient’s quality of life [3,4]. The ureteric stent symptom questionnaire (USSQ) is a validated and reliable instrument to evaluate the impact of different types of stents commonly used in urology [13]. 

The so-called “stent-related symptoms” range from mild discomfort, dysuria, and hematuria, to more significant morbidities, such as UTI, reflux, mal-positioning, fracture, and encrustation [7]. In addition to all of these, a DJ stent is also known to have a significant impact on sexual function in both genders [14]. This aspect is rarely talked about, especially in certain cultures. It results in psychological disturbance, thereby deeply influencing the patient’s quality of life [3].

We evaluated the effect of routine ureteric stenting on female sexual function. We used the standard, validated FSFI questionnaire in English and Urdu, to specifically document the level of dysfunction in sexually active women. All participants were given the choice to fill the form in either language. A significant decline in the total FSFI scores at four weeks after DJ stent placement was observed. It is as yet unclear as to how the DJ stent affects sexual function. One possibility is the presence of bothersome lower urinary tract symptoms resulting in secondary deterioration of sexual function [15].

The results of our study are comparable with the very few other studies conducted in other parts of the world. No similar study has been conducted to look at the effect of DJ stent insertion on women in our part of the world, and our results shall have a significant impact on patient counseling in Pakistan. Our study is also unique in that it made a comparison of FSD between two separate patient cohorts (stented versus non-stented), at three points in time.

In a cohort study conducted by Fawzi et al. in Indonesia, a significant decline in sexual function in a single-stented group of both men and women was observed [16]. Bolat et al. [17] found no correlation between URS and sexual dysfunction in their study where the International Index for Erectile Function (IIEF) for men and FSFI for women were used at one month and three months after the procedure. They reported dysfunction at one month, which showed improvement at three months, and hence concluded that the difference was most likely due to DJ stent [7]. We obtained similar results from our study, as the non-stented women (Group B) who only underwent URS reported no sexual dysfunction at any point in time. Eryildirim et al. have reported similar results in both male and female patients [11]. A notable difference in both IIEF and FSFI scores was reported before and after the installation of DJ stents. Sighinolfi et al. also obtained similar results; however, they evaluated patients before and approximately four weeks after the DJ stent insertion [3].

Our study has some limitations. The reported sample size consisted of a small group of women, and this may have induced bias. The main reason for so many participants dropping out of the study was because of the sensitive nature of this subject in our region. Secondly, any dysfunction due to the stent material and design was not evaluated, as we used only one type of stent in the stented group. More extensive studies need to be done to look at this aspect in further detail in association with the type of stents, and the effect of menopause or other physiologic conditions having an impact on female sexual function. Nevertheless, our preliminary analysis provides valuable insights into a subject that is usually neglected in our part of the world. Looking at the sparse amount of literature available on this “taboo” subject, we believe our results shall contribute to improved patient counseling in this part of the world.

There is a return to baseline sexual function after stent removal by three months, as indicated by our results and that of most of the reported series. Our findings indicate that women with stents were affected more when compared with the women who did not get a DJ stent; however, there was a return to baseline function three months post-procedure. This indicates that the dysfunction is temporary in nature but persistent throughout the stent-indwelling time. However, patients should be informed about this possible outcome during the general preoperative counseling.

## Conclusions

Based on our findings, the use of a DJ stent after an endourological procedure causes temporary sexual dysfunction. There is a need to standardize indications for stenting following URS or RIRS, with particular emphasis on shorter operative times and minimal ureteric trauma. In cases where stenting is unavoidable, patients need to be informed in advance about this adverse effect as well.
